# Oral Lichen Planus and Lichenoid Lesions in Sjogren's Syndrome Patients: A Prospective Study

**DOI:** 10.1155/2019/1603657

**Published:** 2019-05-07

**Authors:** Raouaa Belkacem Chebil, Yassine Oueslati, Marwa Marzouk, Fatma Ben Fredj, Lamia Oualha, Nabiha Douki

**Affiliations:** ^1^Oral Medicine and Oral Surgery, Dental University, Monastir, Tunisia; ^2^Internal Medicine, Medical University, Sousse, Tunisia

## Abstract

**Objective:**

The aim of this study was to investigate the prevalence and characteristics of oral lichen planus (OLP) and oral lichenoid lesions (OLL) in Sjogren's syndrome (SS) patients.

**Patients and Methods:**

A prospective clinical study was conducted at the Department of Oral Medicine and Oral Surgery in Sahloul Hospital, Sousse, from January 2012 to June 2018. The patients involved in this study were diagnosed with Sjogren's syndrome according to the AECG (American-European consensus group) diagnostic criteria. Among these patients, we searched for those affected by OLP or OLL as determined by the WHO (World Health Organisation) classification of 2003. Clinical variables such as age, sex, medical conditions and medications, type of SS (primary or secondary), clinical form of OLP, and treatment were analyzed. The assessment of the results was performed using SPSS software.

**Results:**

We evaluated 30 patients (27 females and 3 males) diagnosed with SS (24 had primary SS) with a mean age of 55 years and 11 months (±11,714). Overall, 9 patients had oral lesions (30%). Two patients had OLP associated with secondary SS (25%). Primary Sjogren's syndrome patients had 6 OLP lesions and one erythematous lichenoid lesion. OLP was erosive in eight patients, among them two had vulvo-vaginal-gingival syndrome. OLP lesions showed improvement in symptoms after topical or general corticosteroids treatment, while OLL showed improvement only under antibiotic treatment.

**Conclusion:**

The results of our analysis suggest that patients with SS have 30% prevalence of OLP and OLL. This possible association shows the importance of screening for oral dryness in patients with OLP or OLL. Treatment includes topical or general corticosteroids for erosive forms associated or not with topical antifungal treatment to treat or prevent oral candidiasis.

## 1. Introduction

Sjögren's syndrome (SS) is a systemic autoimmune disease characterized by marked reduction of exocrine glandular secretions and by its progression to lymphocytic infiltration and destruction [1].

This condition may occur in isolation or in association with organ-specific autoimmune diseases, such as thyroiditis, primary biliary cirrhosis, or cholangitis. In these cases, the disease is referred to as primary Sjögren's syndrome (PSS). However, the term “secondary Sjögren's syndrome (SSS)” is used when the disease is associated with another systemic autoimmune disease, such as rheumatoid arthritis, systemic lupus erythematosus (SLE), scleroderma, or dermatomyositis [2].

The etiopathogenesis is still poorly elucidated. But most probably, it is related to epithelial cells deregulation leading to immune cells attraction. All the epithelial tissues may be attacked even though this pathologic process is more important in the salivary and lacrimal glands [2].

This exocrinopathy often results in dryness of the mouth and eyes, fatigue, and joint pain. The pleiotropic features of the disease are the result of a wide activation of both inflammation as well as innate and adaptive immune pathways that lead to a chronic deregulation of T and B cells. Diagnosis of SS is often made at a late stage due to the delay of its symptoms.

Recently, great efforts have been made to search for reliable biomarkers to ameliorate the diagnostic algorithm [3].

Xerostomia could contribute to an accelerated destruction of the mucosal epithelium, as saliva normally has mechanical, antibacterial, and antimycotic roles in protecting the oral mucosa. As a result, atrophic lesions of the mucosa are a common observation in cases of Sjogren's syndrome and other salivary gland diseases. These epithelial changes can occur in both the skin and mucous membranes in cases of lichen planus.

Oral lichen planus is a chronic inflammatory disease of unknown etiology that affects the oral mucosa. It is well documented that the disease represents a cell-mediated immune response. The diagnostic criteria for oral lichen planus (OLP) are based on clinical and histopathologic features. SS and OLP are two chronic inflammatory diseases similar in many aspects. These two pathologies are frequently associated with autoimmune disorders, and both have immune dysfunction. T-lymphocyte infiltration is also found in SS and OLP patients. Atrophy, fibrosis, fatty degeneration in the acini, changes in the ductal structure, and lymphocytic infiltration are common changes observed on minor salivary gland biopsies from patients with OLP, suggesting the presence of a salivary gland disease [4].

These data suggest that both diseases are also related in a pathological manner.

The aim of this study was to investigate, through a prospective clinical study, the prevalence and characteristics of oral lichen planus (OLP) and lichenoid lesions (OLL) in Sjogren's syndrome (SS) patients.

## 2. Materials and Methods

A prospective clinical study was conducted at the Department of Oral Medicine and Oral Surgery in Sahloul Hospital from January 2012 to June 2018. The patients involved in this study were diagnosed with SS according to the 2002 AECG (American-European consensus group) diagnostic criteria [5]. Diagnosis of PSS requires four of the six following criteria including either positive histopathology or positive autoimmune serology: subjective symptoms of oral and/or ocular dryness; oral and/or ocular signs (determined by salivary and lacrimal gland functions); positive histopathology and/or positive autoimmune serology. Diagnosis of PSS is made if the patient has 3 of the 4 positive objective responses: oral signs, ocular signs, positive histopathology, or positive autoantibodies [6].

Secondary SS is retained for patients having ocular symptoms or oral symptoms together with any two positive objective responses from ocular signs, oral signs, or histopathology with an underlying autoimmune diagnosis [6].

Among these patients, we searched for those affected by oral lichen planus (OLP) or lichenoid lesions (OLL) as determined by the WHO (World Health Organisation) classification of 2003. We excluded patients with epithelial dysplasia or having lesions close to silver amalgam restorations [7].

Pertinent data were gathered including the patients' demographics (sex and age), medical history, medications, SS diagnostic criteria values, type of Sjogren's syndrome (primary or secondary), clinical form of OLP, its location, *Candida* infection, biopsy results, and treatment modality. The assessment of the results was performed using SPSS software.

## 3. Results

### 3.1. Demographics

In total, 30 patients with well-characterized Sjogren's syndrome were evaluated. The mean age of these patients was 55 years and 11 months (SD ± 11,714). Twenty-seven (90%) patients were females.

### 3.2. SS Classification Criteria

Minor salivary gland biopsy was conducted for all the patients. 63.33% (19 patients) of these biopsies were positive (stage 3 or 4). Autoantibodies were obtained in 11 patients (36.66%), with the presence of a positive anti-SSA or anti-SSB autoantibody serum test in 7 (23.33%) patients.

Seven patients had a positive anti-SSA autoantibody titer, and 6 (20%) had a positive anti-SSB autoantibody titer.

Overall, diagnosis of PSS was made for 23 (76.66%) patients while 7 patients were diagnosed with the secondary form.

### 3.3. Oral Lesions

Overall, 9 patients had oral lesions (30%). Two patients had oral lichen planus (OLP) associated with secondary Sjogren's syndrome. Primary Sjogren's syndrome patients had 6 oral lichen planus (OLP) lesions and one lichenoid lesion (Table 1).

**Figure 1:**
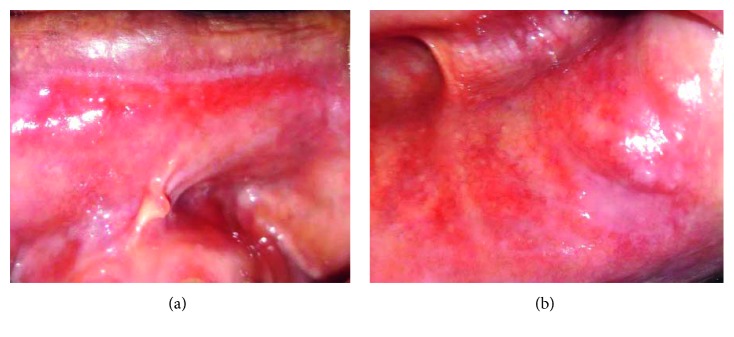
Oral erythematosus lichenoid lesion of the lips' inner surfaces of patient 7 (absence of reticular pattern). (a) Upper lip. (b) Lower lip.

**Figure 2:**
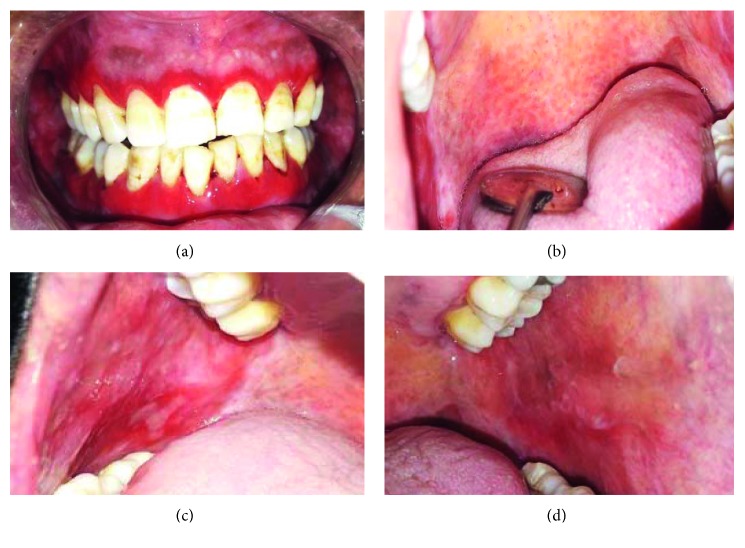
Vulvo-vaginal-gingival syndrome: oral lesions (patient 5). (a) Erosive gingivitis. (b) Soft palate involvement with extension to esophageal region. (c), (d) Erosive lesions of the inner surface of the cheeks.

The most frequent locations of OLP were the inner surfaces of the cheeks, lips, and gingiva (Table 2). Lichenoid lesion was located in the inner surface of the lips, and it was resistant to conventional treatment (Figure 1). Three patients suffered from vitamin D deficiency. Two patients presented vulvo-vaginal-gingival syndrome in association with cutaneous lesions with esophageal involvement and negative autoantibodies anti-SSB in one case (Figure 2). This patient had a history of hysterectomy 5 years earlier. All the oral lesions were treated by oral cavity conditioning and topical corticosteroids (SOLUPRED®) associated with topical antifungal treatment in two cases (oral candidiasis). Tapering doses of oral corticosteroids over 4 weeks in addition to topical treatment with steroid ointment (DERMOCORT®) on the vaginal mucosa was indicated for vulvo-vaginal-gingival syndrome. This treatment showed an improvement of the erosive lesions. Lichenoid lesion of the inner surface of the lip was ameliorated following topical mouthwash and antibiotic treatment (CLAMOXYL®) (Table 2).

## 4. Discussion

To the best of our knowledge, this study provides the first evaluation of the prevalence of OLP and OLL among primary and secondary SS patients.

Recently, the American College of Rheumatology (ACR) and the European League Against Rheumatism (EULAR) have validated criteria for the classification of PSS.

On the basis of the listed classification criteria (Table 3), diagnosis of a primary Sjogren's syndrome is defined as having a score 4 or more. Positive serologic results for anti-SSB/La antibodies in the absence of anti-SSA/Ro antibodies are not specific and are no longer considered to be a criterion for the diagnosis [2]. Since the classification criteria for SSS are not yet validated, we adopted in our study the 2002 AECG classification.

The pathophysiology of PSS implies the activation of mucosal epithelial cells, possibly from viral stimulation. This leads to the activation of the immune systems with the secretion of autoantibodies. Recent studies have suggested the presence of activated CD8 T cells in the blood and glands [2].

Similarly, patients with oral lichen planus suffer from the consequences of immune dysfunction with T-lymphocyte (CD8 T cells) infiltration.

It has been reported that autoimmune diseases are observed in 33% of SS patients including primary biliary cirrhosis, hypothyroidism, rheumatoid arthritis, Graves' disease, discoid lupus, coeliac disease, dermatomyositis, and scleroderma [8]. The same associations were noted for oral lichen planus which is frequently observed in patients with autoimmune diseases such as myasthenia gravis, hypothyroidism, rheumatoid arthritis, graft host disease, irritable bowel syndrome, ulcerative colitis, psoriasis, thymoma, lupus erythematosus, and coeliac disease.

The mean age of SS patients found in our study was 55 years, which corresponds to the mean ages of SS and OLP lesions.

Likar-Manookin et al. studied the prevalence of oral lesions having autoimmune etiology (OLAIE) in patients with primary Sjogren's syndrome and found that lichen planus and recurrent aphthous stomatitis were the two most common oral lesions [9]. Sistig et al. reported that salivary immunoglobulin levels increase in both OLP and SS [10].

These data suggest that both diseases are related in a pathological manner. In their study, Likar-Manookin et al. found that 7% of patients with PSS had oral lichen planus lesions [9]. In our study, we found that, from 23 patients with PSS, 7 (30.43%) had OLP. Previous studies indicated that roughly 1% of the general population has OLP [11]. Likar-Manookin et al. reported that 9/190 (4.7%) OLP patients were also found to have PSS [9]. Prospectively, we evaluated the prevalence of PSS and glandular lymphocytic infiltrate on OLP patients who underwent minor salivary gland biopsies. We found that, from 25 OLP patients, seven had PSS and 4 had lymphocytic infiltrate with a focus score <1. The higher prevalence of SS disease among OLP patients found in our study is due to systematic sialometric analysis and minor salivary gland biopsy for patients presenting for OLP or OLL.

To verify if the oral lesions described in our study are related to lichenoid reactions to medications, we compared the drug classes between the patients with OLP or OLL versus those without oral lesions. The patients presenting no OLP were more medicated than those having these lesions.

In our study, we noted that lichenoid lesion was located in the inner surface of the lips and was resistant to conventional treatment. This oral lichenoid lesion is described as mucositis of the upper lip and gingiva and is considered by some authors as a clinical subtype of lichenoid lesions. The main features making it a distinct clinical entity are the location (the mucosa of the upper lip and gingiva), the clinical appearance which shows intense erythema, and resistance to conventional treatment with topical and systemic glucocorticosteroids. A microbial factor is strongly speculated in the pathogenesis [12].

The association of this lesion with PSS in our patient suggests that a reduced salivary flow rate may enhance this microbial etiology. Georgakopoulou et al. suggests that the combination of clarithromycin and prednisolone may be effective for the treatment of this lichenoid lesion [13].

Two patients presented with lichen planus of the vulva and vagina with desquamative gingivitis which is described as the vulvo-vaginal-gingival syndrome. Only a few previous reports of this syndrome and even fewer with esophageal involvement were found. In their case series study, Rauschecker et al. found that esophageal lichen planus typically occurs in older women with longstanding dysphagia and often develops in the absence of extraesophageal sites of disease. This may be explained by the fact that many patients have the disease confined to the esophagus before the development of oral or cutaneous lesions, resulting in esophageal strictures and dysphagia [14]. Upper gastrointestinal endoscopy reveals multiple ulcers in the esophageal mucosa [14]. For our patient (Figure 2), esophageal lesion appeared one year after oral and vaginal erosions. Then, anti-SSB antibodies were negative, and this patient had a history of hysterectomy while presenting the most serious condition among all the OLP patients studied. Likar-Manookin et al. found three potential risk factors for having both PSS and oral lesions of autoimmune etiology: anti-SSB negativity, irritable bowel syndrome (IBS), and use of hormone replacement or oral contraceptives [9].

All the OLP lesions were erosive. The treatment of these symptomatic forms included oral cavity conditioning and topical steroid rinses. Systemic steroids were indicated for vulvo-vaginal-gingival syndrome (tapering doses of oral corticosteroids CORTANCYL 30 mg over 4 weeks). Topical antifungal treatment was prescribed for two patients. Oral candidiasis is a frequent complication of SS, and it is also a common side effect of these medications [15]. Therefore, prophylactic antifungal medications should be associated with topical steroids. Antifungal treatment should be started before performing biopsy if the lesion is complicated by oral candidiasis because of histologic analysis difficulties. The status of serum 25-hydroxyvitamin D in OLP patients, when investigated (5 Patients), was reduced consistently. Jie Du et al. conducted an experimental study on the role of 1,25(OH)_2_D_3_ amelioration of oral lichen planus. They suggested that 1,25(OH)_2_D_3_ plays an anti-inflammatory role in OLP, and they concluded that vitamin D supplement may be a potential strategy for OLP management [16].

Pedersen et al. [17] conducted a preliminary study on the effects of bovine colostrum-containing oral hygiene products in patients with primary SS and oral lichen planus. Their results suggested beneficial effects on the oral symptoms as well as the general health.

## 5. Conclusion

Two hypotheses could be retained. OLP could be triggered by SS or the same immunological and genetic factors could have played a role in the development of both diseases.

These findings highlight the importance of screening patients having oral lichen planus for Sjogren's syndrome by sialometric analysis and minor salivary gland biopsy, even if they are not specifically referred for that purpose. Treatment involves topical or general corticosteroids for erosive forms associated or not with topical antifungal treatment to treat or prevent oral candidiasis.

This study has an important limitation. It was performed on a small sample size and was geographically restricted to Tunisia. Thus, generalization of the results should be made with caution. Data were not obtained from a specialized center in internal medicine which may be considered as a recruitment bias.

## Figures and Tables

**Table 1:** Oral lichen planus and lichenoid lesions in primary and secondary Sjogren's syndrome (SSS) patients.

	OLP	OLL	Total
PSS	6	1	23
SSS	2	0	7
Total	8	1	30

**Table 2:** Sex, age, autoantibodies, medical condition, oral lesion characteristics, and treatment modality.

Sex/age	Autoantibodies (anti-SSA/anti-SSB)	Medical condition	Sjogren's syndrome	Oral lesions	Location	Treatment
1. ♀/44	Negative	Diabetes	Primary	Erosive OLP	(i) Gingiva (ii) Inner surface of the cheeks	(i) Oral cavity conditioning (ii) Topical mouthwash (Solupred) (iii) Dermocort (iv) Topical antifungal treatment

2. ♀/52	Negative	(i) Psoriasis (ii) Rheumatic psoriasis (iii) Vitamin D deficiency (iv) Allergy to Co, Cr, Ni	Secondary	Erosive OLP	(i) Inner surface of the cheeks (ii) Inner surface of the lips	(i) Oral cavity conditioning (ii) Topical mouthwash (Solupred) (iii) Vitamin D supplementation

3. ♂/65	Not mentioned	(i) Gastric ulcer (ii) Anemia	Primary	Erosive OLP	(i) Dorsal side of the tongue (ii) Inner surface of the cheeks (iii) Inner surface of the lips	(i) Oral cavity conditioning (ii) Topical mouthwash (Solupred)

4. ♀/40	(i) Negative (ii) Positive (iii) Rheumatoid factor	(i) Anemia (ii) Rheumatoid Arthritis	Secondary	Erosive OLP	(i) Inner surface of the cheeks (ii) Inner surface of the lips	(i) Oral cavity conditioning (ii) Topical mouthwash (Solupred)

5. ♀/51	Anti-SSA + anti-SSB -	(i) Hypertension (ii) Hyperlipidemia (iii) Vitamin D deficiency (iv) Hysterectomy	Primary	Erosive OLP Vulvo-vaginal-gingival syndrome	(i) Gingiva (ii) Soft palate (iii) Esophageal mucosae (iv) Inner surface of the cheeks (v) Skin lesions (vi) Vulvar and vaginal lesions	(i) Oral cavity conditioning (ii) Topical mouthwash (Solupred) (iii) Vitamin D supplementation (iv) General treatment with corticosteroids

6. ♀/60	Not mentioned	Vitamin D deficiency	Primary	Erosive OLP	Inner surface of the lips and cheeks	(i) Oral cavity conditioning (ii) Topical antifungal treatment (iii) Topical mouthwash (Solupred) (iv) Mouthwash (Chlorhexidine) (v) Vitamin D supplementation

7. ♀/60	Not mentioned	Vitamin D deficiency	Primary	Erythematosus OLL	Inner surface of the lips	(i) Oral cavity conditioning (ii) Topical antifungal treatment (iii) Topical mouthwash (Solupred) (iv) Mouthwash (Chlorhexidine) (v) Vitamin D supplementation (vi) Antibiotherapy (CLAMOXYL®).

8. ♀/62	Not mentioned	Diabetes	Primary	Erosive OLP	(i) Inner surface of the cheeks (ii) Skin lesions (iii) Vulvar and vaginal lesions	(i) Oral cavity conditioning (ii) Topical mouthwash (Solupred)

9. ♀/56	Not mentioned		Primary	Erosive OLP	Inner surface of the lips	(i) Oral cavity conditioning (ii) Topical mouthwash (Solupred) (iii) General treatment with corticosteroids

^*∗*^OLP: oral lichen planus; ^*∗*^OLL: oral lichenoid lesion. ♀: female; ♂: male.

**Table 3:** 2017 ACR-EULAR classification criteria for primary Sjögren's syndrome [2].

Item	Score
Focus score of ≥1	3
Presence of anti-SSA antibodies	3
SS ocular staining score of ≥5	1
Schirmer's test of ≤5 mm per 5 min	1
Unstimulated whole salivary flow of ≤0.1 ml per min	1
Total score	9

^*∗*^SS: Sjögren's syndrome.

## Data Availability

Previously reported (data type) data were used to support this study and are available at (doi or other persistent identifier). These prior studies (and datasets) are cited at relevant places within the text as references.

## Additional Points

*Areas of Uncertainty*. Recent pathophysiological studies have shown a number of similarities between primary Sjögren's syndrome and systemic lupus erythematosus SLE, which suggests that primary Sjögren's syndrome may represent a form of SLE affecting the mucosa [2]. Oral lichen planus is frequently associated with SS and SLE, and it has clinical and histopathological similarities with the oral lesions of SLE. Could OLP also represent a form of SLE affecting the mucosa?
